# Ciliogenesis and the DNA damage response: a stressful relationship

**DOI:** 10.1186/s13630-016-0040-6

**Published:** 2016-06-22

**Authors:** Colin A. Johnson, Spencer J. Collis

**Affiliations:** Section of Ophthalmology and Neurosciences, Wellcome Trust Brenner Building, Leeds Institute of Molecular Medicine, St. James’s University Hospital, Leeds, LS9 7TF UK; Genome Stability Group, Department of Oncology and Metabolism, Academic Unit of Molecular Oncology, Medical School, University of Sheffield, Beech Hill Road, Sheffield, S10 2RX UK

**Keywords:** Cilia, DNA damage response, Cell cycle, Stress signalling, Ciliopathies, Cancer

## Abstract

Both inherited and sporadic mutations can give rise to a plethora of human diseases. Through myriad diverse cellular processes, sporadic mutations can arise through a failure to accurately replicate the genetic code or by inaccurate separation of duplicated chromosomes into daughter cells. The human genome has therefore evolved to encode a large number of proteins that work together with regulators of the cell cycle to ensure that it remains error-free. This is collectively known as the DNA damage response (DDR), and genome stability mechanisms involve a complex network of signalling and processing factors that ensure redundancy and adaptability of these systems. The importance of genome stability mechanisms is best illustrated by the dramatic increased risk of cancer in individuals with underlying disruption to genome maintenance mechanisms. Cilia are microtubule-based sensory organelles present on most vertebrate cells, where they facilitate transduction of external signals into the cell. When not embedded within the specialised ciliary membrane, components of the primary cilium’s basal body help form the microtubule organising centre that controls cellular trafficking and the mitotic segregation of chromosomes. Ciliopathies are a collection of diseases associated with functional disruption to cilia function through a variety of different mechanisms. Ciliopathy phenotypes can vary widely, and although some cellular overgrowth phenotypes are prevalent in a subset of ciliopathies, an increased risk of cancer is not noted as a clinical feature. However, recent studies have identified surprising genetic and functional links between cilia-associated proteins and genome maintenance factors. The purpose of this mini-review is to therefore highlight some of these discoveries and discuss their implications with regards to functional crosstalk between the DDR and ciliogenesis pathways, and how this may impact on the development of human disease.

## Background

Recent work from several groups has strengthened the ever-expanding functional links between the DNA damage response (DDR) and ciliogenesis. Given that both the DDR and primary ciliogenesis are stress response mechanisms that are inextricably linked to the cell cycle (see below), then these findings are perhaps not too unexpected in the context of their biological function. Furthermore, centrioles, which can help govern genome stability in proliferating cells through correct microtubule organisation and accurate chromosome segregation, also form the basal body of primary cilia within quiescent cells. However, defects in DDR/genome stability factors are traditionally associated with inherited cancer-predisposing diseases syndromes, whereas patients with ciliopathies do not have an increased risk of cancer development. This makes recent findings that mutations in some DDR proteins are causal for a subset of human ciliopathies all the more intriguing. The following sections will therefore give a brief overview of the recently discovered genetic and functional links between DDR and ciliogenesis. We highlight key proteins identified to date that have dual roles in these biological processes.

### The DNA damage response and genome stability

DNA within cells is damaged on a daily basis from both exogenous sources e.g. UV radiation from the sun or carcinogens within tobacco smoke, and from endogenous sources e.g. metabolic by-products, errors introduced during DNA replication, or by chromosome segregation defects during mitosis [1]. In order to maintain genomic integrity and to minimise the accumulation of potentially pro-mutagenic lesions within the genome, sophisticated molecular mechanisms have evolved to resolve the numerous daily lesions that can occur within a cell, e.g. DNA breaks (single and double-stranded), base and sugar damage to the DNA backbone, DNA and DNA-protein cross-links, base-pair mismatches incorporated during DNA replication and alkylation lesions on various sites of the DNA [1, 2]. These processes involve highly specialised sets of proteins and pathways that mediate the detection and repair of specific lesions, but often possess overlapping functions between the many different DNA repair pathways [1, 2]. The detection and subsequent repair of DNA damage are coordinated with the cell cycle through a series of complex regulatory and feedback mechanisms known collectively as cell cycle checkpoints [3–5]. Such checkpoints can be activated at various stages of the cell cycle process to allow time for DNA lesions to be resolved before progressing to the next stage of the cell cycle [5]. This is vital for maintaining the sequence integrity of the genome, as failure to carry out these process can lead to potential pro-mutagenic lesions being ‘fixed’ during replication and passed on to daughter cells during mitotic segregation of the chromosomes [4, 6]. If the damage to the genome is beyond a cell’s ability to adequately repair it, then cell death mechanisms are triggered that act as a final fail-safe to prevent the propagation and passage of potentially pro-mutagenic lesions to daughter cells [3–5]. The collective term for the detection and subsequent repair of potentially pro-mutagenic DNA lesions is the “DNA damage response” (DDR), which, together with pro-apoptotic mechanisms, acts as a critical barrier to the development of cancer [1, 7–9]. The importance of an intact DDR in combating tumourigenesis is perhaps best demonstrated by the numerous human cancer-predisposing disease syndromes that are a consequence of underlying mutations in DDR factors [1, 10, 11]. Additionally, it is well established that there is an increased risk of either breast or colorectal cancer in individuals with mutations in specific DDR factors e.g. BRCA1/2 and MSH2, MSH6 etc. [1, 10, 11]. Mutations in genes encoding a plethora of DDR factors can also lead to a range of other human inherited or sporadic disorders with several overlapping clinical phenotypes [1, 10]. The most common overlapping clinical trait associated with mutations in such factors is congenital microcephaly, potentially due to defects in neurogenesis during the developing embryo [12]. The rapid cell expansion that takes place during this process is susceptible to DNA damage [13], and also requires accurate asymmetric cell division. As such, mutations in proteins that have important functions in DNA replication, DNA repair, centrosome maintenance, microtubule regulation, and mitosis have all been shown to be causal for several human microcephalic disorders [12] (see Table 1 for some examples).

**Table 1:** Examples of centrosomal proteins that are mutated in human microcephalic or ciliopathy disorders, and have known functional roles in DDR or genome integrity

Protein	Protein function	Associated disease/clinical features	Possible role in the DDR?
CEP63	Promotes accurate duplication of centrioles and assembly/integrity of the mitotic spindle	Seckel syndrome (SCKL6); microcephaly, short stature/dwarfism and delayed speech development	A substrate of DDR kinases and over-expression can lead to supernumerary centrosomes and DNA damage. MRNA over-expression in a cohort of neuroblastomas was associated with MYCN amplification and poor patient prognosis
CEP152	Cooperates with Cep63 to promote accurate centriole duplication	Primary microcephaly (MCPH9) and Seckel syndrome (SCKL5); microcephaly, moderate cognitive impairment and mild behavioural disorders	Chromosomal defects observed in SCKL5 patients with potential heightened replicative stress and DNA damage, although this has not been extensively studied to date
CEP164	Localises to the basal body to promote ciliogenesis	Nephronophthisis-related ciliopathies; retinal degeneration (including blindness) and seizures	Is a substrate of DDR kinases, localises to nuclear foci in response DNA damage and facilitates cellular responses to UV irradiation
CEP290	Required for the integrity of many centriolar satellites, microtubule organisation and ciliogenesis	Bardet–Biedl (BBS14), Joubert (JBTS5), Meckel (MKS4) and Senior–Løken syndrome (SLSN6) syndromes; clinical features include retinal and renal abnormalities, brain malformation, dysplastic kidneys and nephronophthisis, respectively	No overt increased cancer risk associated with BBS14, JBTS5, MKS4 or SLSN6. Many identified frameshift mutations identified in various cancers, with a 10 % mutation frequency in endometrial cancers (TCGA)
MCPH1	Required for cell cycle checkpoints in response to DNA damage, and preventing chromosome condensation (PCC)	Primary microcephaly; microcephaly, growth retardation/dwarfism, cognitive impairment, radiation sensitivity and PCC evident in patient cells	Prevents genome instability by facilitating cell cycle checkpoints in response to DNA damage. Deleted in several cancers including breast and ovarian cancer (TCGA). Increased incidence of lymphomas in MCPH1^−/-^ mouse models
NEK8	Promotes cell cycle progression from G2-M and ciliogenesis by regulating Cyclin A–CDK activity	Nephronophthisis (NPHP9) and renal-hepatic-pancreatic dysplasia (RHPD2); dysplasia in the kidney as well as in the liver and pancreas	Promotes ATR-mediated cellular responses to replication stress to prevent to accumulation of DNA damage. Over-expressed in some breast tumours
PCNT	Microtubule organisation, accurate chromosome segregation and ciliogenesis	Microcephalic osteodysplastic primordial dwarfism type II (MOPDII); microcephaly, short stature and bone abnormalities	Facilitates cellular responses to DNA damage through interaction with the DDR effector kinase CHK1

### DDR factors and centrosomes

The centrosome acts as the major site of microtubule nucleation and organisation in both interphasic and mitotic cells, and forms the basis of the basal body during ciliogenesis (see below). It consists of two orthogonally positioned, cylindrically shaped structures known as centrioles, which are surrounded by an electron-dense matrix termed the pericentriolar material (PCM) and acts as an organised scaffold that facilitates protein recruitment to the centrosome. Associated with the PCM are numerous particles termed centriolar satellites, which contain many components of the PCM and other centrosomal proteins [14–17]. The formation, maturation and duplication of centrosomes are regulated in unison with the cell cycle [16]. As such, defects in cell cycle progression, e.g. following the induction of DNA damage, can lead to changes in the composition and architecture of centriolar satellites and give rise to centrosome duplication errors [18–21]. As the duplication of centrosomes occurs during the G1/S-phases of the cell cycle, cells experiencing persistent DNA damage and checkpoint activation and/or replication stress which prolongs the time spent within S-phase, can give rise to abnormal centrosome duplication called supernumerary centrosomes [21–23]. Additionally, it was recently shown that some centriolar satellites form an interactome together with centrosomal proteins to promote CDK2 activity and efficient centriolar duplication [24].

Given the important roles of the centrosome within the cell and functional overlap with DDR (see above), it is perhaps not too surprising that defects in centrosome-associated factors that function in DDR processes give rise to a range of human inherited disorders [11, 25, 26] that include several microcephalic disorders and ciliopathies (Table 1). This includes examples of clinicopathological overlap between ciliopathy and microcephaly patients [27], as well as mutations in the microtubule-regulating protein CENPF that are associated with both ciliopathy and microcephaly disorders [28]. In addition, there is a long-standing connection between supernumerary centrosomes, genome instability and cancer development and/or progression, since supernumerary centrosomes are a common hallmark of cancer cells [25, 29–34]. A functional consequence of abnormal centrosome number within the context of cancer was recently highlighted by the demonstration that centrosome amplification can lead to cell adhesion changes that can help drive the invasive phenotypes associated with metastatic cancer cells [35]. However, it is interesting to note that even given the dual role of many centrosome-associated proteins within ciliogenesis (Table 1), and that cilia-associated signalling pathways are often dysregulated in cancers, there is not an overt association between ciliopathy and cancer risk (discussed below).

Functional links between the DDR and centrosomes have been previously inferred by the centrosomal localisation of several DDR factors including the DNA repair proteins BRCA1, BRCA2, PARP1 and NBS1; the DDR signalling kinases ATM, CHK1 and CHK2; and the cell cycle checkpoint and transcriptional regulator TP53 [36, 37]. However, it must be noted that antibody cross-reactivity in these studies cannot be excluded without thorough reagent validation [38, 39]. More convincing mechanistic insights into biological function come from the observation that the E3 ubiquitin ligase BRCA1 ubiquitylates gamma-tubulin at centrosomes, which is important for restricting centrosome over-duplication during S and G2 phases of the cell cycle [40] that, in turn, is regulated by NBS1 and the upstream DDR-associated kinase ATR [41]. The DDR effector kinase CHK1 was initially reported to also localise to the centrosome [36, 42], but this was subsequently determined to be through a non-specific interaction of the CHK1 antibody cross-reacting with the centrosomal protein CCDC151 [39]. It is therefore not currently clear how CHK1 may contribute to the mechanism of centrosome amplification by NBS1 and BRCA1 functions which are both capable of activating CHK1 in response to DNA damage and/or replication stress [43, 44]. However, CHK1 function has since been shown to be important for regulating expansion of the PCM [45], a process that has been shown to affect the growth of daughter centrioles [46]. Additionally, CHK1 together with the centrosomal protein MCPH1 (Table 1) can control mitotic entry [39, 47]. Interestingly, changes in MCPH1 expression have been associated with both breast and ovarian cancer grade, which may be a consequence of increased cell division in higher-grade tumours [48, 49]. Changes in either centriole duplication in S-phase due to PCM expansion or inappropriate cell cycle timing could therefore be mechanisms by which alterations in CHK1 function could impact centrosome integrity, although further studies to address these issues are clearly needed.

Interactions between centrosome-associated and DDR proteins can also occur in response to exogenous stress. For example, the centrosomal and ciliogenesis-promoting protein CEP164 (Table 1) is phosphorylated by the DDR-associated kinases ATM and ATR in response to several genotoxic stresses where it helps establish a G2/M damage checkpoint and regulate cell division processes [50]. CEP164 has also been shown to re-localise to sites of UV-induced damage, and is required for efficient cellular responses to UV-induced DNA damage [51]. However, it is presently not clear if this is a specific response to UV, or a more general response to replication-blocking lesions and/or induction of p38-mediated stress signalling pathways. It is interesting to note that the core centriolar factor centrin 2 has both centriolar localisation and a major nuclear component. The latter functionally responds to UV-induced DNA damage and physically interacts with XPC to promote efficient repair of UV-induced DNA lesions [52–54]. Recent studies suggest that ATM can also act as a versatile protein kinase during cytoplasmic signalling processes [55], and ATM may therefore have a “non-canonical DDR” ciliary role that maintains genome stability and mediates cellular responses to various other cellular stresses. Indeed, there are a number of centrosome-associated proteins that are known or predicted in vivo substrates of the DDR-associated kinases ATM, ATR and DNA-PKcs, which include centrosomal and ciliary proteins such as ninein, PCM1 and INPP5E [56]. Another example of a centrosome protein that is a direct substrate of DDR kinases is CEP63 (Table 1), which is phosphorylated by ATM and ATR to promote mitotic spindle assembly [57], and has been shown to regulate centriole duplication [58, 59], potentially through centrosomal CDK activity [60]. However, unlike CEP164, a direct role for CEP63 in cellular response to DNA damage is yet to be elucidated. Additionally, although not a directly associated DDR kinase, the kinase Aurora A regulates mitotic entry and exit as well as cilium disassembly [61]. One of Aurora A’s substrates is the mitotic kinase PLK1 which can also promote cilia disassembly and has been shown to function in cell cycle checkpoint recovery following DNA damage [62, 63]. Consistent with these findings is work from several groups linking the APC, which co-ordinates mitotic progression in response to DNA damage and replication stress, to ciliogenesis [64, 65]. Finally, we have recently demonstrated that some centriolar satellite proteins have dual roles in promoting ciliogenesis and preventing the accumulation of DNA damage within the cell [20, 66].

The examples highlighted here (see Table 1 for additional examples) demonstrate both physical and functional interactions between DDR centrosomal proteins, many of which control ciliogenesis. The majority of interplay between the DDR and centrosome proteins involves either regulating centrosome duplication through the cell cycle, or regulating accurate timing of mitotic entry through the spindle pole body. Such crosstalk between these processes may therefore be important for driving faithful cell division during early development, as shown by the example of microcephalic disorders, and may also be linked to uncontrolled cell division during tumour progression and/or development. Further elucidation of the functional connectivity between these cellular processes should provide new insights into a number of human inherited and sporadic disorders (Table 1).

### The cellular role of mammalian cilia

Primary cilia are microtubule-based organelles that sense and transduce extracellular signals on many cell types during the G_1_/G_0_ phase of the cell cycle [67, 68]. Cilia have a complex ultrastructure with compartmentalisation of molecular components that combine in functional modules. The loss or mutation of these components can disrupt ciliary functions such as the control of protein entry and exit from the cilium, regulation of signalling cascades and control of the cell cycle. In particular, the ciliary transition zone has been suggested as a hub that mediates and integrates paracrine signalling during embryonic development and tissue morphogenesis, including the SHH, WNT and Notch signalling pathways [69–72]. A common mechanism for regulating these pathways appears to be the discrete compartmentalisation of signalling components to the cilium. As a paradigm for other pathways, Smo, the co-receptor and transducer for SHH, translocates into and then activates GLI transcription factors within the cilium [73]. Canonical WNT/β-catenin signalling is also constrained by compartmentalisation of the WNT signalling component Jouberin, ensuring the translocation of β-catenin away from the nucleus and into the cilium [74]. In turn, Notch signalling is proposed to be a modulator of ciliary SHH signalling by regulating the ciliary translocation of Smo [75]. More recently, the mTOR [76, 77], Hippo [78–80], TGFβ [81] and PDGF [82] signalling pathways have all been shown to be regulated through ciliary-dependent mechanisms, with diverse consequences on cell proliferation and size, differentiation, autophagy, apoptosis and tumourigenesis. It is presently unclear to what extent any of the ciliary-related signalling pathways modulate DDR, although a recent study has suggested that the Notch1 receptor binds to and negatively regulates the activity of the DDR-associated kinase ATM [83], and may be part of an interactome with other DDR-associated factors [84]. It will therefore be interesting to determine what effect any further connections between the Notch1 receptor and ATM have on ciliogenesis. From these studies, the reported connections between centrosomal and ciliary proteins with DDR link the processes of cilium biogenesis and disassembly with the mitotic and S-phase checkpoint pathways that monitor failures in DNA replication and chromosome transmission. The disruption of these ciliary processes may therefore permit dysregulated cell proliferation, a hallmark of all cancers. Conversely, recent work has led to the growing recognition that alterations of replication timing and progression, leading to replication stress and activation of DDR, are features of some renal ciliopathies [85, 86].

Systems biology approaches have revealed a widespread role for spliceosome proteins and other mRNA-processing factors in preventing DNA damage, which in some cases was caused by aberrant RNA–DNA structures [87]. Many of the same spliceosome and mRNA-processing components, including those mutated in inherited forms of the retinal degeneration condition retinitis pigmentosa, were also identified in a recent reverse genetics screen for genes and pathways regulating ciliogenesis [88]. Loss of primary cilia has also been observed in tumours of many cancers, including breast cancer [89] and renal cell carcinomas [90], prompting suggestions that the cilium may be a “tumour suppressor organelle”. For example, familial adenomatous polyposis (FAP or Gardner syndrome), an inherited Wnt-dependent cancer, may be mediated by a ciliary-dependent mechanism [91]. However, the mechanistic details to explain these observations remain unknown, so it is unclear if cilia loss contributes to or is merely a consequence of the nuclear events of replication stress and activated DDR.

It is also important to appreciate that signalling pathways have multiple roles in maintaining normal adult tissue homeostasis that are distinct to developmental signalling during embryogenesis. The role of primary cilia in developmental SHH signalling is well established, but this pathway also regulates the survival and proliferation of tissue progenitor and stem cell populations [92]. These mitogenic roles can explain why abnormal activation of the canonical SHH signalling pathway, either through activating mutations in pathway components or by ligand production in an autocrine mechanism, predisposes to cancer in many different tissues, including medulloblastoma, glioblastoma and basal cell carcinoma [93–95]. Whether primary cilia are essential for the mitogenic roles of SHH is presently unclear. For example, tumourigenesis caused by activating mutations in the SHH co-receptor Smo is decreased if cilia are ablated, whereas cilia loss increased tumourigenesis caused by activated GLI2, a transcriptional effector of SHH signalling [96]. However, the complex mitogenic roles of SHH provide one explanation of why there is no apparent increase in cancer incidence in ciliopathy patients.

### Emerging genetic and functional links between the DDR and primary cilia

It has recently been shown that in proliferating cells, several centriolar satellite proteins are re-structured following exogenous stresses such as UV that, in turn, repress inhibitory signals and facilitate ciliogenesis [97]. Similarly, stress-induced autophagy can affect the composition of centriolar satellites to promote ciliogenesis [98]. Conversely, stress signalling through the primary cilium helps regulate autophagy by promoting the formation of the autophagosome [99]. We have also demonstrated that some centriolar satellite proteins act to promote ciliogenesis as well as genome stability [20, 66], which may in part be through regulation of the composition of the centrosome and centriole duplication through CDK2 activity [24]. Stress signals emanating from DNA damage can be either intra- or intercellular through a variety of mechanisms involving cell–cell contacts and/or extracellular signalling collectively known as ‘bystander effects’ [100]. Interplay between the DDR and primary cilia may therefore involve both internal functional interactions between DDR and centriolar/basal body proteins, as well as external signals from neighbouring cells. The last few years have seen emerging functional links between autophagy and DDR, where autophagy facilitates cell fate following DNA damage and also helps prevent genome instability to combat tumourigenesis [101, 102]. Interestingly, autophagy processes may also be responsive to DNA damage-induced bystander effects, facilitating both intra- and intercellular stress signalling. This complex interplay between these cellular stress-responsive mechanisms has potential implications for ciliopathies and microcephalic disorders, as well as for cancer [24, 101].

In addition to the examples given above that demonstrate physical and functional connections between DDR and centrosomal proteins, work from several groups has revealed direct genetic and functional links between DDR and ciliogenesis (Tables 1, 2). As mentioned above, the pro-ciliogenesis centrosomal protein CEP164 is regulated by DDR kinases and promotes cellular responses to UV-induced DNA damage [50, 51]. More recently, homozygous recessive mutations in CEP164 were shown to be causal for a subset of nephronophthisis-related ciliopathies, with mutant zebrafish models exhibiting both ciliopathy phenotypes and inefficient responses to DNA damage [103]. Furthermore, this study also showed that NPHP10 (also known as SDCCAG8), which usually resides at centrosomes, re-localised to nuclear foci in response to DNA damage [103], and a subsequent study has suggested that deficiency in NPHP10 (either in cell models or in cells derived from knock-out mice) leads to elevated levels of DNA damage and cell cycle checkpoint activation [104]. Consistent with an established functional role for some of the NEK kinase family members in both DDR and ciliogenesis [105], it was recently reported that the ciliopathy-associated kinase NEK8 (Table 1) is important in controlling cellular responses to replication stress through the DDR kinase ATR and limiting CDK activity to suppress DNA break formation [106]. What is more surprising, given the non-overlapping clinical phenotypes of NEK8-associated ciliopathies and ATR-associated Seckel syndrome patients, is that cells expressing a ciliopathy-associated kinase mutant NEK8 had an increase in DNA damage and cell cycle defects, and that the kidneys of NEK8 mutant mice accumulated DNA damage [106]. Furthermore, the centrosomal protein CEP290, mutated in a range of ciliopathies including Joubert syndrome, has also been implicated in the regulation of DNA replication stress and DDR (Table 1), suggesting that chronic replication stress may be a key driver in the development of some ciliopathies [85, 86]. Similar to the NEK8 study, cells expressing mutant CEP290 also had inappropriate CDK activity. Tissue-specific replication stress in certain genetic backgrounds may therefore be a common mechanism that drives the development of a subset of ciliopathies, and suggests that CDK may be a potential therapeutic target for such diseases [85, 86].

**Table 2:** Examples of established DDR-associated proteins currently linked to ciliogenesis and/or ciliopathy disorders

Protein	Protein function in DDR	Role in ciliogenesis	Associated human disease
ATMIN	Co-factor of the PIKK ATM that promotes its activation and cellular responses to DNA damage and replication stress. Originally designated ASCIZ for ATM/ATR-substrate Chk2-interacting Zn(2 +)-finger protein	Through its role as a transcription factor, it regulates WNT signalling that is important for correct morphogenesis of the developing lung and kidney of mice	None associated to date, although recessive homozygous mutations in its binding partner ATM lead to the developmental and cancer predisposition syndrome Ataxia-telangiectasia (A-T)
ATR	Phosphoinositide 3-kinase related kinase (PIKK) central to co-ordinating cellular responses to replication stress by activating appropriate cell cycle checkpoints and homologous recombination mediated DNA repair processes	Localises to the basal body in the developing eye of mice where it promotes ciliogenesis. Zebrafish genetic models of Seckel syndrome exhibit impair sonic hedgehog signalling and ciliogenesis	Splicing defects and compound heterozygous mutations in ATR are causal for the growth retardation disease Seckel syndrome. ATR is therefore also known as SCKL1
FAN1	Structure-specific nuclease involved in the repair of DNA interstrand cross-links. Associated with the Fanconi Anaemia DNA repair pathway	None presently known	Biallelic mutations in FAN1 are causal for a subset karyomegalic interstitial nephritis and germ-line mutations in humans associated with colorectal cancer
MRE11	Key nuclease involved in resection of DNA double-strand breaks to promote their repair; mainly through homologous recombination processes	None presently known	Mutations in MRE11 are causal for the rare cancer-predisposition disease ataxia-telangiectasia-like disorder-1 (ATLD1). Homozygous recessive mutations are causal for a subset of nephronophthisis-related ciliopathies. Often over-expressed in cancers, and may promote increased error prone micro-homology end-joining DNA repair process that gives rise to genome instability
ORC1	Forms part of the origin recognition complex that initiates DNA replication. Also localises to the centrosome to regulate centrosome duplication and ciliogenesis	Meier–Gorlin syndrome (MGS1); microcephaly, short stature/dwarfism and skeletal abnormalities. Note that mutations in related DNA replication proteins ORC4, ORC6, CDT1 and CDC6 all cause Meier–Gorlin syndrome	Controls DNA replication, which is responsive to DNA damage through DDR signalling. Oncogene-induced replication stress can be a source of tumourigenic mutations, although it is not clear if reduced ORC1 function could facilitate this process
VCP/p97	Recruited to DNA breaks where it facilitates the recruitment of other DDR factors and their ubiquitylation-mediated regulation. Also involved in cellular responses to replication stress	Recently shown to regulate the E3 ligase-mediated ubiquitylation of UBXN10 to control the trafficking rates of cilia by the intraflagellar transport B complex	Mutations in VCP/p97 have been implicated as causal for inclusion body myopathy with Paget disease of bone and frontotemporal dementia (IBMPFD)

It is intriguing that the same study identifying CEP164 mutations as causative for a subset of nephronophthisis-related ciliopathies also identified causative mutations in MRE11 (Table 2). MRE11 interacts stoichiometrically with RAD50 and NBS1 (forming the so-called MRN complex) to facilitate key functions of DNA repair processes [103]. Specifically, germ-line mutations in either NBS1 or MRE11 give rise to the cancer-predisposing inherited disorders Nijmegen breakage syndrome and ataxia-telangiectasia-like disorder (ALTD), respectively [107, 108]. Furthermore, MRE11 has been shown to function as a barrier to tumourigenesis [109, 110], and inherited heterozygous mutations in MRE11, NBS1 or RAD50 are associated with a low-intermediate penetrance risk of breast cancer [111–113]. It is presently unclear how or why specific mutations in MRE11 in particular can give rise to ciliopathies. This raises interesting questions about whether mutations in other members of the key DDR-associated MRN complex (MRE11-RAD50-NBS1), mutations which cause inherited cancer syndromes [114], may also be causative for other renal-retinal ciliopathies. Perhaps even more surprising was the recent discovery that mutations in the Fanconi Anaemia and cancer-associated nuclease FAN1 (Table 2; [115–119]) could be causative for a subset of karyomegalic interstitial nephritis-type ciliopathies [120]. As this enzyme is involved in the repair of DNA lesions that block DNA replication, the study suggested that defective nuclease activity within certain organs could drive cellular senescence following increased exposure to genotoxins (perhaps arising from a heightened active metabolism). This may be a similar scenario to the proposed heightened replication stress observed in the kidneys of both CEP290 and NEK8-deficient mice (see above). Although this may be a mechanism by which FAN1 mutations can give rise to ciliopathies, the underlying biology may be more complicated, especially given that phenotypes associated with karyomegalic interstitial nephritis-type ciliopathies are not evident in patients with Fanconi anaemia (FA). Such phenotypic discrepancy may also be, in part, due to the redundancy within the pathways that function to resolve DNA replication-impeding lesions [121].

In addition to these genetic studies, several groups have also uncovered functional links to ciliogenesis for proteins traditionally associated with the DDR. An example of this is the recent finding that ATR localises to the basal body in mouse photoreceptor cells (Table 2), and is important for ciliogenesis during the developing eye [122]. ATR is also required for ciliary-related Sonic hedgehog signalling in vitro and in vivo, but appears to be largely dispensable for ciliogenesis, in a role that is distinct from its function in DDR and replication [123]. Another finding is that mutations in DNA replication licensing factors such as ORC1 (Table 2), were causative for the microcephalic disorder Meier–Gorlin syndrome (MGS) and were also shown to affect ciliogenesis through impaired SHH signalling [124]. The AAA-ATPase protein VCP/p97, which regulates the localisation of several DDR factors at DNA damage sites [125], has been shown to be required for ciliogenesis (Table 2), when it may carry out similar functions in regulating E3 ligase-mediated ubiquitylation of proteins at the basal body [126]. Finally, the protein ATMIN, a binding partner of the key DDR kinase ATM and also important for cellular responses to replication stress [127, 128], has also been shown to be important for ciliogenesis during morphogenesis of both the lungs and kidneys in developing mice through its ability as a transcription factor to regulate WNT signalling [129, 130]. Collectively, these studies demonstrate both genetic and functional links between DDR and ciliogenesis (Table 2).

### The human primary cilium and cancer

Contrary to these recent discoveries involving DDR-associated factors in human ciliopathies is the general observation that an increased risk or incidence of cancer is not generally associated with human ciliopathies. Exceptions include Birt–Hogg–Dubé syndrome and Von Hippel–Lindau syndrome that are both inherited renal cancer disorders with some clinical features of ciliopathies [131, 132]. Furthermore, although patients with polycystic kidney disease have benign renal cysts as a consequence of a cell overgrowth phenotype, they do not have an increased risk of developing cancer, and may in fact have an overall reduced cancer risk compared with non-affected individuals [133, 134]. It is not clear why this may be the case, but it has been suggested that a coincident increased rate of cell death through either apoptotic and/or autophagy mechanisms might help reduce cancer risk in affected individuals. A similar phenomenon has been reported for genetic reduction of ATR activity limiting the tumour growth of P53-deficient tumours in mice [135], although an increased risk of cancer in some Seckel syndrome patients has been reported, with at least one of these having a causative genetic defect in the *ATR* gene [136, 137]. Interestingly, it has recently been suggested that increased replication stress, similar to that often seen in cancers due to oncogene activation, is a phenotype associated with a subset of ciliopathies, such as CEP290-associated Joubert syndrome [85, 86]. Thus, it may be that a certain level of tolerance to heightened replication stress is needed in order to drive more tumourigenic phenotypes associated with DDR-related diseases, which is not selected during the development of the majority of human ciliopathies.

The studies briefly highlighted here provide compelling evidence of ever-expanding genetic and functional links between DDR and ciliogenesis pathways. However, the discrepancies between the phenotypes of DDR-associated cancer-predisposing syndromes and ciliopathies (Tables 1, 2) do not fit with our current limited knowledge of how these two pathways could be connected. This may reflect the functional impact each pathway has within both developing and differentiated tissue, as well as how normal or aberrant pathway function may affect both pre-cancerous lesions and transformed cells.

## Concluding remarks

The purpose of this mini-review is to highlight emerging links between cellular responses to DNA damage and ciliogenesis. Although some of these studies provide more mechanistic insight into this functional overlap than others, we are still some way from fully understanding the intricate interplay between DDR and ciliogenesis factors. Such links were initially striking given the established role the DDR plays in preventing tumourigenesis and the lack of any increased cancer risk in the majority of human ciliopathy patients. However, it is becoming clear from recent genetic and functional-based studies that a subset of DDR and ciliogenesis factors have dual roles in maintaining genomic integrity and primary cilia biology. The majority of this duality appears to stem from the necessity of a cell to regulate centrosome duplication and mitotic spindle integrity, with several DDR proteins localising to the centrosome and/or regulating cell cycle progression and, in turn, centriole duplication events. Additionally, several centriolar satellites help maintain appropriate centrosome structures and microtubule integrity to limit the accumulation of post-mitotic DNA damage. Finally, aberrant mitogenic signals (potentially through a common mechanism of inappropriate CDK activity) can give rise to replication stress which can, in turn, lead to aberrant centrosome duplication and maturation processes. As such, heightened replication stress may be a common source of disrupted centrosome function in cancer, and aberrant cilia function in ciliopathies.

The majority of human cells are ciliated with the cilium acting as a signalling hub for several interconnected stress response pathways, which are in constant communication with the DNA damage response pathways and cell cycle regulators. Recent discoveries demonstrating that autophagy and ciliogenesis can co-regulate each other, and that autophagy is responsive to oxidative stress/DNA damage and can regulate DNA repair processes, further draw links between primary cilia and the DDR. Such functional interplay has implications for human disease, which is highlighted by the recent discoveries of mutations in proteins, traditionally thought to be solely involved in DNA repair processes, being causative for a subset of human ciliopathies with degenerative diseases of the kidney and retina. With the advent of next-generation sequencing of larger clinical cohorts, it will also be interesting to see if additional DDR factors and autophagy factors are implicated in ciliopathies, and if dysregulation in any cilia-associated factors is associated with an increased risk of cancer development and progression. Indeed, given the young age and small cohort of current ciliopathy patients with causative mutations in either *FAN1* or *MRE11*, it is too early to determine if these patients have an increased risk of developing cancer. Given that mutations in both these proteins can give rise to various cancers (see above), one may predict that these ciliopathy patients may have a heightened risk of developing cancer compared with the general population and some other ciliopathy cohorts. For these conditions, pathogenic mechanisms of replication stress leading to DNA damage, concomitant with or upstream of primary cilia function, are an area of exciting future research. Finally, since ciliogenesis and replication stress are potentially reversible with small molecule approaches, these findings also reveal new therapeutic intervention opportunities as possible treatment regimes for these diseases.

## Abbreviations

APC: anaphase-promoting complex
ATM: ataxia-telangiectasia mutated
ATMIN: ATM interactor
ATR: ATM-related
BRCA1: breast and ovarian cancer susceptibility protein 1
BRCA2: breast and ovarian cancer susceptibility protein 1
CDK: cyclin-dependent kinase
CEP164: centrosomal protein 164KDa
CHK1: checkpoint kinase 1
DDR: DNA damage response
FA: Fanconi anaemia
FAN1: FANCD2/FANCI-associated nuclease
G1: growth phase 1 of the cell cycle
G2: growth phase 2 of the cell cycle
M: mitotic phase of the cell cycle
MGS: Meier–Gorlin syndrome
MRE11: meiotic recombination 11 homolog A
mTOR: mammalian target of rapamycin
NEK8: NimA-related kinase 8
NPHP10: nephronophthisis-related ciliopathy protein 10
NBS: Nijmegen breakage syndrome
PARP1: poly (ADP-Ribose) polymerase 1
PCM1: pericentriolar material 1
PDGF: platelet-derived growth factor
S: DNA synthesis phase of the cell cycle
SHH: sonic hedgehog signalling pathway
SMC1: structural maintenance of chromosomes 1
Smo: smoothened
TGFβ: transforming growth factor β
TP53: tumour suppressor protein 53 kDa
VCP: valosin-containing protein
WNT: wingless-related integration site
